# Reverting iodine avidity of radioactive-iodine refractory thyroid cancer with a new tyrosine kinase inhibitor (K905-0266) excavated by high-throughput NIS (sodium iodide symporter) enhancer screening platform using dual reporter gene system

**DOI:** 10.18632/oncotarget.24159

**Published:** 2018-01-11

**Authors:** Ji Min Oh, Senthilkumar Kalimuthu, Prakash Gangadaran, Se Hwan Baek, Liya Zhu, Ho Won Lee, Ramya Lakshmi Rajendran, Chae Moon Hong, Shin Young Jeong, Sang-Woo Lee, Jaetae Lee, Byeong-Cheol Ahn

**Affiliations:** ^1^ Department of Nuclear Medicine, Kyungpook National University School of Medicine and Hospital, Daegu 41944, Republic of Korea; ^2^ BK21 Plus KNU Biomedical Convergence Program, Department of Biomedical Science, Kyungpook National University School of Medicine and Hospital, Daegu 41944, Republic of Korea

**Keywords:** anaplastic thyroid cancer, sodium iodide symporter, tyrosine kinase inhibitor, radioactive-iodine therapy, high-throughput screening

## Abstract

Radioactive-iodine (RAI) therapy is typically unprevailing as anaplastic thyroid cancer (ATC) management, owing to the decrease in the endogenous sodium iodide symporter (NIS) expression. Therefore, new strategies for NIS re-induction are required to improve the efficacy of RAI therapy in ATC. In this study, we developed a novel high-throughput NIS enhancer screening platform using a dual reporter gene system to identify a potent tyrosine kinase inhibitor (TKI) and selected a new hit compound, K905-0266 TKI. The effects of K905-0266 TKI treatment was validated as RAI accumulation, changes in signalling pathway related to thyroid pathogenesis, and cytotoxicity of RAI depending on re-induction of endogenous NIS expression in ATC. Furthermore, we evaluated enhancement of NIS promoter and therapeutic efficacy of RAI in ATC tumour xenograft mice. After K905-0266 TKI treatment, the expression of endogenous NIS was significantly increased, while phosphorylated-ERK was decreased. In addition, the thyroid-metabolising protein expressions were upregulated and increased of RAI accumulation and its therapeutic effects in ATC. Moreover, K905-0266 TKI increased therapeutic efficacy of RAI in ATC tumour *in vivo*. In conclusion, we successfully established a novel high-throughput NIS enhancer screening platform to excavate a NIS enhancer and identified K905-0266 TKI among TKI candidates and it's proven to increase the endogenous NIS expression and therapeutic efficacy of RAI in ATC. These findings suggest that a novel high-throughput NIS enhancer screening platform is useful for selecting of NIS promoter enhancers. In addition, K905-0266 TKI can be used to re-induce endogenous NIS expression and recover RAI therapy in ATC.

## INTRODUCTION

Anaplastic thyroid cancer (ATC) is a rare form of undifferentiated thyroid cancer, which arises from differentiated thyroid cancer through the accumulation of genetic defects [1]. Although the incidence rate is relatively low among all thyroid cancers, it is characterised by aggressiveness, rapid progression, poor prognosis, and high mortality rates. In addition, metastasis of ATC occurs rapidly to distant sites such as regional lymph nodes, lungs, bones, and brain via haematogenous spreading [2]. Multimodal treatment including surgery and chemo-radiotherapy has been applied for the management of ATC but resulted in minimal survival benefit [3].

Sodium iodide symporter (NIS) is an intrinsic transmembrane glycoprotein, which transports two sodium cations (Na^2+^) with an iodide anion (I^−^) in the basolateral membrane of thyroid follicular cells. The ability to accumulate iodide has been widely used as a therapeutic strategy to target cytotoxic radioactive iodine (RAI) to unresectable thyroid cancers [4, 5]. ATC fails to adequately maintain original features of thyroid follicular cells, such as iodine uptake and thyroglobulin synthesis; hence, these cells show suppressed NIS expression and decreased iodine avidity [6, 7]. Therefore, ATC patients display poor prognosis and a high recurrence rate because of un-differentiation characters and resistance to RAI, which is followed by the loss of iodine uptake function [8]. Hence, the recovery of iodine avidity by re-differentiation of the cell and re-induction of endogenous NIS expression in ATC is necessary for effectiveness of RAI therapy [9].

The pathogenesis of ATC is closely associated with aberrant activation of mitogen-activated protein kinase (MAPK) pathway, which is frequently caused by the BRAF^V600E^ mutation [10]. MAPK pathway has a critical role in the development and progression of thyroid cancer and its activation results in silencing of iodide-metabolising genes and impairment of NIS in thyroid cancer [11]. Therefore, the blockade of MAPK pathway may lead to the reversion of unresponsiveness RAI therapy to sensitive RAI therapy. Several preclinical studies have gained partial success with the use of various inhibitors related to MAPK pathway [12–14]. In clinical trials, BRAF^V600E^ inhibitor (dabrafenib) and MAPK inhibitor (selumetinib) have been reported to enhance iodide uptake in thyroid cancers [15, 16]. At present, several on-going preclinical and clinical studies using various inhibitors related to phosphatidylinositol-3-kinase-protein kinase B (PI3K-Akt) and MAPK pathway are aiming to discover stronger re-differentiating agents.

Screening system techniques such as high-throughput screening (HTS), high-content screening, surface plasmon resonance, chemical chip, and computer virtual screening have been used to identify new compounds against pharmacological targets [17–20]. HTS technology is an efficient approach to discover new compounds against a specific target and has been most widely applied for the development of targeted agents [21, 22].

In this study, a novel high-throughput NIS enhancer screening platform using bioluminescence dual reporter gene system to evaluate NIS promoter activity was developed and applied to select a potent NIS enhancer among tyrosine kinase inhibitor (TKI) libraries. Furthermore, we adopted K905-0266 TKI as a potent NIS enhancer and investigated the induction of endogenous NIS expression mediated by RAI therapy in ATC cells.

## RESULTS

### 8505C cells stably expressing dual reporter gene system were successfully established

**Figure 1:**
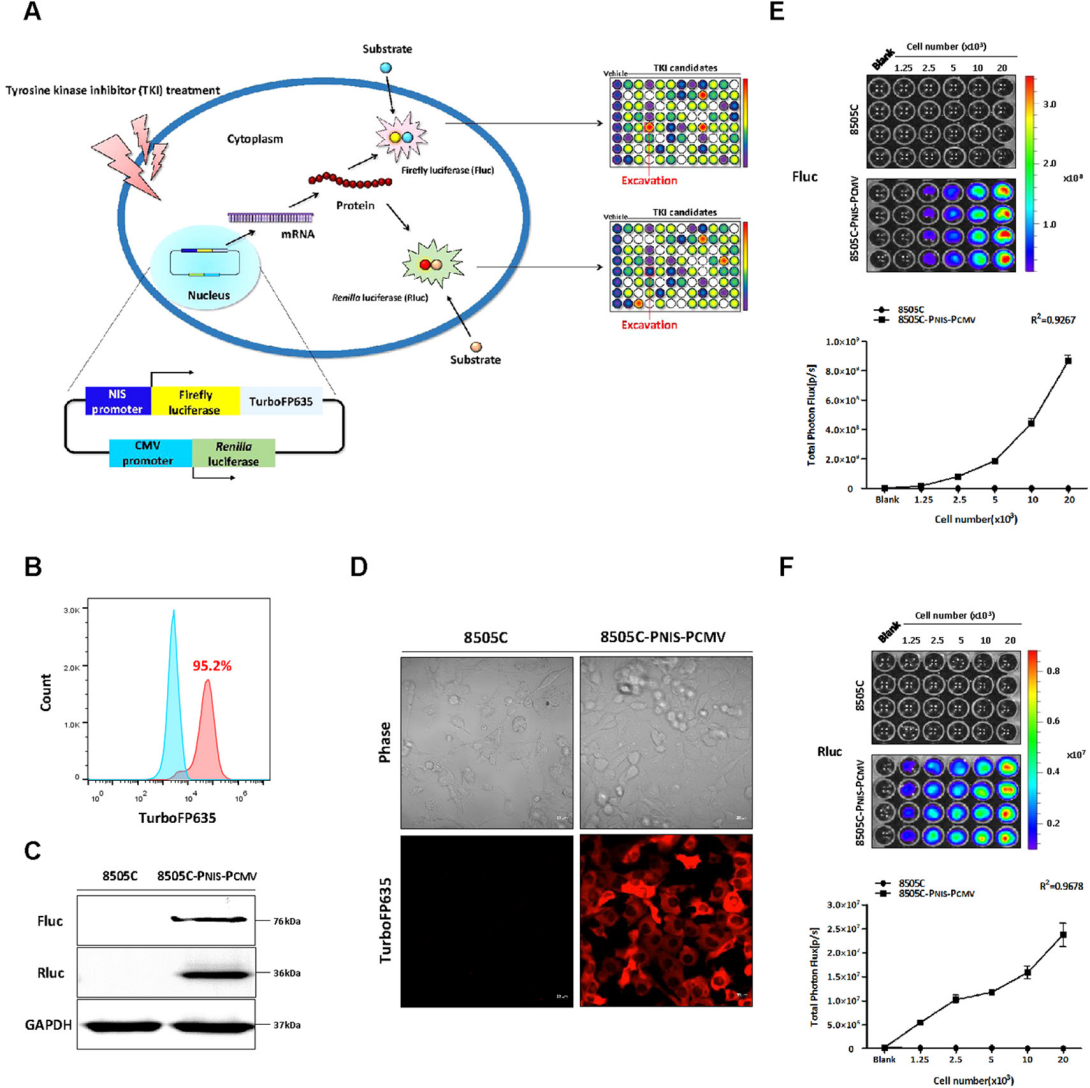
Schematic illustration of dual reporter gene system and establishment of a stable cell line expressing dual reporter gene system **(A)** The plasmid DNA expressing dual reporter gene system was constructed. Fluc and TurboFP635 were expressed under the control of NIS promoter, while Rluc was expressed under the control of CMV promoter. **(B)** FACS analysis of 8505C-PNIS-PCMV cells. **(C)** Western blots for the detection of Fluc and Rluc proteins from 8505C and 8505C-PNIS-PCMV cells. **(D)** Confocal microscopy images to detect the expression of TurboFP635 fluorescent protein in 8505C and 8505C-PNIS-PCMV cells. **(E)** Bioluminescence imaging (BLI) and quantitative analysis of Fluc signal activity. **(F)** BLI and quantitative analysis of Rluc signal activity. Data are expressed as the mean ± standard deviation (SD).

To monitor the transcriptional regulation of NIS promoter expression in 8505C-PNIS-PCMV cells, dual reporter gene system expressing both Fluc and TurboFP635 under NIS promoter and Rluc under CMV promoter was designed and constructed (Figure 1A); this vector was named as “pNIS-FL2-TurboFP635-pCMV-Rluc”. The pNIS-FL2-TurboFP635-pCMV-Rluc vector was transfected into 8505C cells and geneticin was used to select the positive clones. Fluorescent-activated cell sorting (FACS) analysis revealed that 95.2% cells were positive for 8505C-PNIS-PCMV (Figure 1B). The expression of the reporter genes in 8505C-PNIS-PCMV cells was identified with western blot analysis of Fluc and Rluc proteins as well as TurboFP635 fluorescence imaging. The expression of Fluc and Rluc proteins was revealed in 8505C-PNIS-PCMV cells but not 8505C cells (Figure 1C). The expression of TurboFP635 protein was also reported to be strong in 8505C-PNIS-PCMV cells, while it was absent in 8505C parental cells (Figure 1D). Furthermore, the functional activities of Fluc and Rluc were tested with Bioluminescence imaging (BLI). BLI signal of both Fluc (Figure 1E) and Rluc (Figure 1F) was significantly increased with an increase in the number of 8505C-PNIS-PCMV cells. Both cell counting kit-8 (CCK-8) assay (Supplementary Figure 2A) and Rluc activity (Supplementary Figure 2B) showed similar results of cell viability.

### High-throughput screening platform approach to excavate a potent NIS enhancer with dual reporter gene system

**Figure 2:**
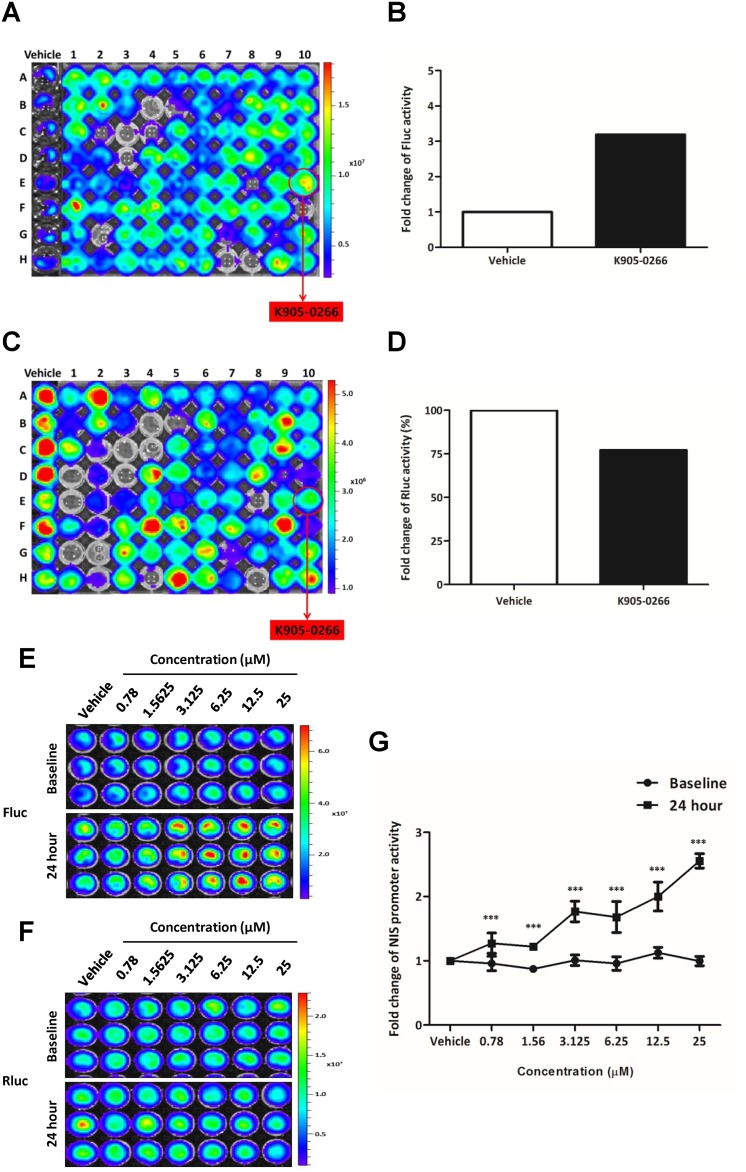
Exploration and excavation of tyrosine kinase inhibitor (TKI) as a potent NIS enhancer (K905-0266) by using high-throughput NIS enhancer screening platform in 8505C-PNIS-PCMV cells **(A)** BLI for Fluc signal activity and selection of K905-0266 TKI showing an increase in NIS promoter activity. **(B)** Quantitative analysis of Fluc signal in cells treated with vehicle control and K905-0266 TKI. **(C)** BLI for Rluc signal activity to evaluate cell viability. **(D)** Analysis of the quantitative comparison of Rluc activity between cells treated with vehicle control and K905-0266 TKI. **(E)** Assessment of the increase in Fluc signal activity in 8505C-PNIS-PCMV cells treated with K905-0266 TKI in a concentration-dependent manner. **(F)** Assessment of Rluc signal activity to evaluate cell viability following treatment of 8505C-PNIS-PCMV cells with K905-0266 TKI. **(G)** Quantitative analysis of NIS promoter activity as the ratio of Fluc to Rluc activity (Fluc/Rluc). The results are indicated as mean ± standard deviation (SD) of experiments performed in triplicates. ^***^P<0.001 (by Student's *t*-test).

To investigate NIS enhancer, 8505C-PNIS-PCMV cells were treated with numerous TKI candidates for 24 hours. The NIS promoter activity evaluated with the quantitative analysis of Fluc BLI signal in 8505C-PNIS-PCMV cells showed a 3.19-fold increase following K905-0266 TKI treatment (Figure 2A, 2B). However, Rluc BLI signal, reflecting cell viability, was 77% upon treatment with K905-0266 TKI as compared with that in the vehicle control (Figure 2C, 2D). Taken together, these results suggest that K905-0266 TKI stimulates the transcription of NIS by increasing the activity of NIS promoter.

### K905-0266 TKI was selected as a NIS enhancer by using the high-throughput NIS enhancer screening platform

8505C-PNIS-PCMV cells were exposed with different concentrations of K905-0266 TKI and assessed the change in NIS promoter activity at multiple time points. As shown in Figure 2E, treatment with K905-0266 TKI gradually increased Fluc signal, which is reflective of NIS promoter activity. The concentration of 12.5 μM showed the highest activity. Compared with the vehicle, Rluc activity was slightly decreased in K905-0266 treatment group (Figure 2F, Supplementary Figure 2). NIS promoter activity normalised by cell viability was calculated using Fluc and Rluc signal intensity. K905-0266 TKI treatment increased NIS promoter activity in 8505C-PNIS-PCMV cells (Figure 2G). Three different concentrations (3.125, 6.25, and 12.5 μM) of K905-0266 TKI were used for the subsequent experiments.

### Expression of endogenous NIS protein following K905-0266 TKI treatment

**Figure 3:**
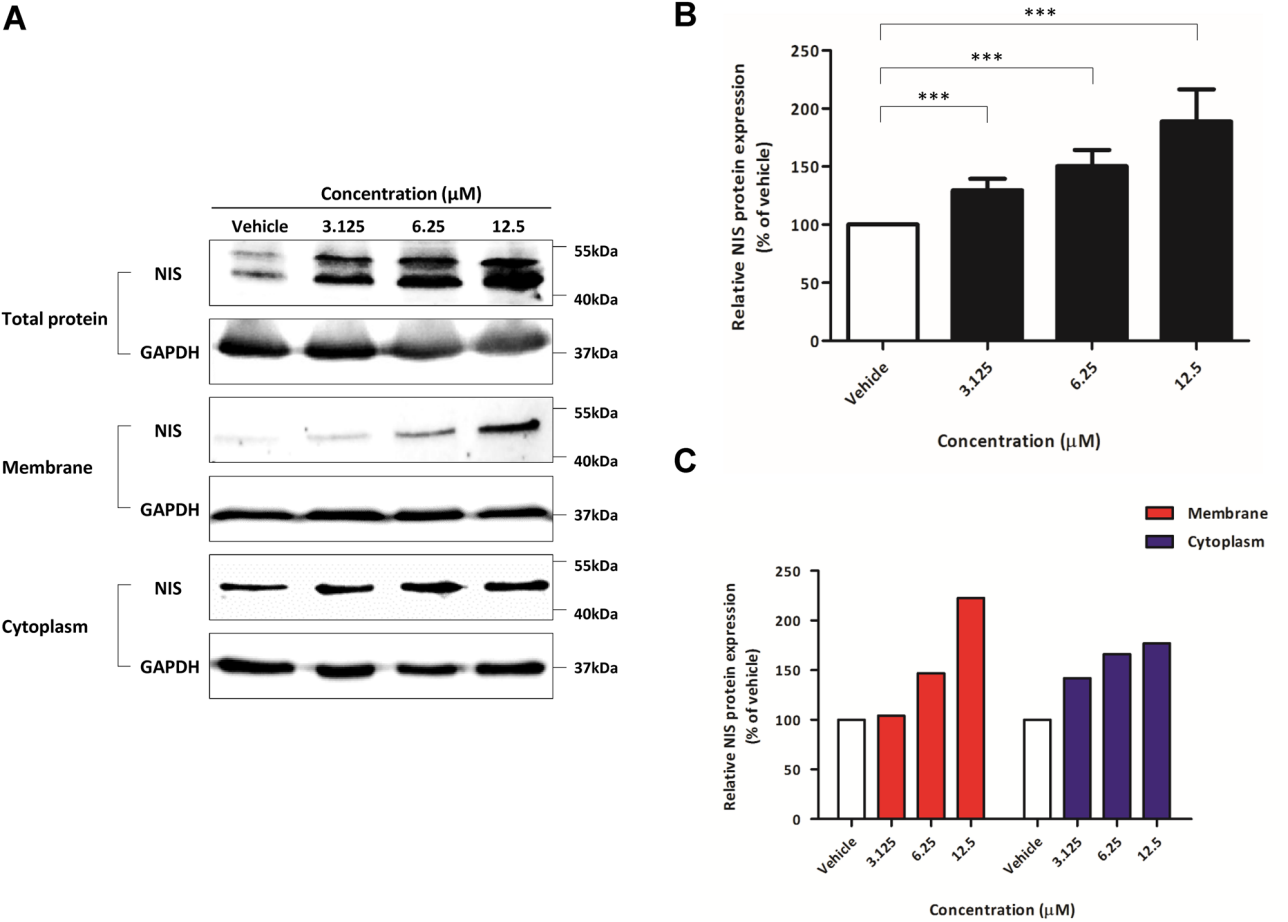
Evaluation of endogenous NIS protein expression promotion, signalling pathways related to re-induction of NIS **(A)** Western blots for NIS protein expression. 8505C cells were exposed to K905-0266 TKI for 24 hours and the total, membrane and cytoplasm proteins were isolated. GAPDH was used as a loading control. **(B)** Quantitative analysis of NIS expression levels from total protein. The results are expressed as mean ± standard deviation (SD) of experiments performed in triplicates ^***^P<0.001 (by Student's *t*-test). **(C)** Quantitative analysis of the expression of both membrane and cytosolic NIS protein following K905-0266 TKI treatment.

Based on the results of BLI signal, the expression of endogenous NIS protein was determined following treatment with K905-0266 TKI. In comparison with the vehicle control, K905-0266 TKI treatment significantly increased the expression of endogenous NIS protein; the most effective dose was 12.5 μM (Figure 3A). The quantitative measurement of the band intensity on the western blot clearly showed a dose-dependent increase in NIS protein expression after K905-0266 TKI treatment (Figure 3B; percentage (%) relative to vehicle intensity 126.80±10.630, 157.404±21.657, and 173.70±31.806 at 3.125, 6.25, and 12.5 μM concentration, respectively). K905-0266 TKI treatment significantly increased NIS protein localization in the cell membrane while uptick changes of cytoplasm NIS protein (Figure 3A, 3C).

### Downregulation of mitogen-activated protein kinase (MAPK) signalling pathway by K905-0266 TKI treatment

**Figure 4:**
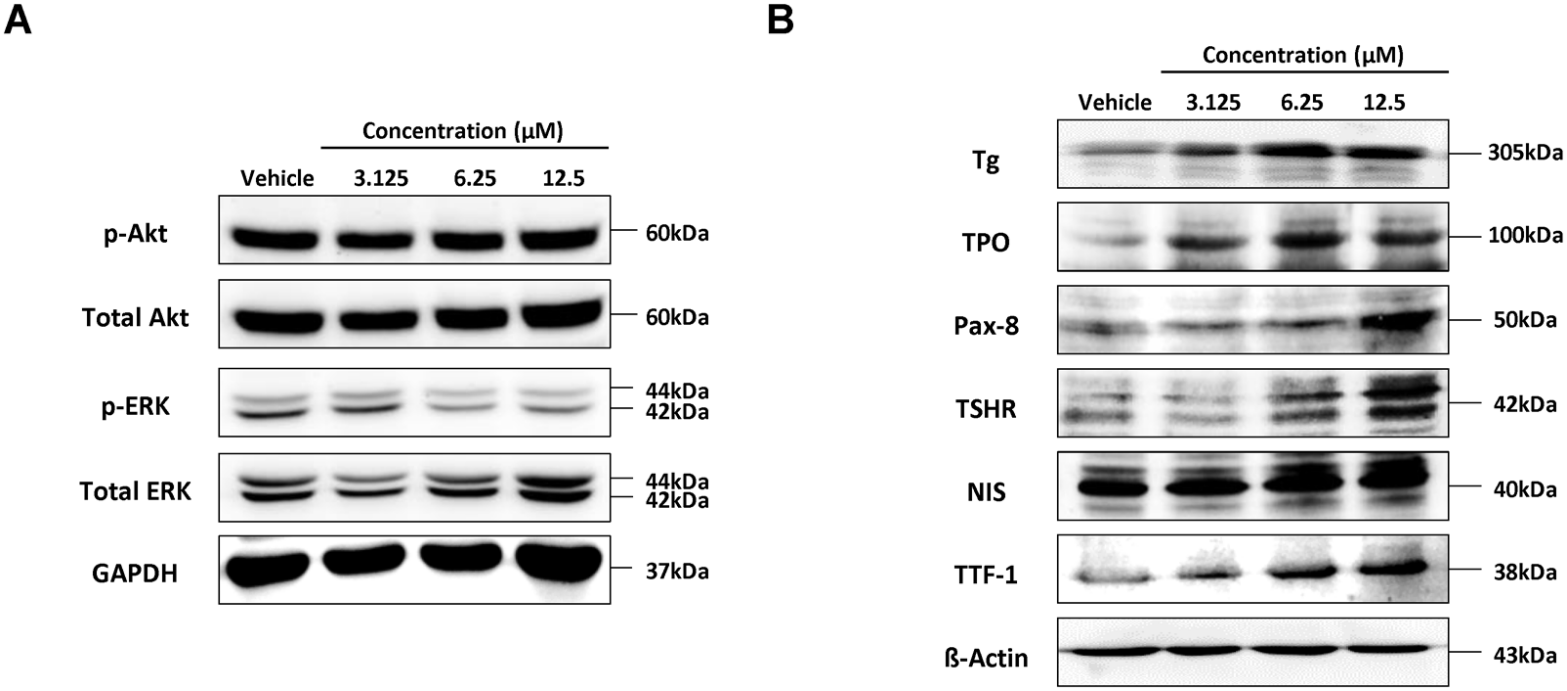
Investigation of signalling pathways related to re-induction of NIS and thyroid-metabolising protein expression following treatment of 8505C cells with K905-0266 TKI Each group was incubated with different concentrations of K905-0266 TKI for 24 hours in 8505C cells. **(A)** Western blot analysis of PI3K-Akt and MAPK signalling pathways. GAPDH was used as a loading control. **(B)** Western blot analysis for thyroid-metabolising protein expression. β-Actin was used as a quality control.

MAPK and PI3K-Akt signalling pathway related to the expression of thyroid-specific genes was assessed. No significant change was observed in the level of phosphorylated Akt following K905-0266 TKI treatment. However, the level of phosphorylated ERK was remarkably downregulated by K905-0266 TKI treatment. These results demonstrated that the enhancement in endogenous NIS protein expression may be related to the inhibition of phosphorylated ERK (Figure 4A).

### Effect of K905-0266 TKI treatment on the expression of TPO, TSHR, Tg, TTF-1, and Pax-8

The factors TPO, TSHR, Tg, TTF-1, and Pax-8 are essential for thyroid follicular cell function. The protein expression of TPO, TSHR, Tg, TTF-1, and Pax-8 was enhanced by K905-0266 TKI treatment (Figure 4B). These results suggested that the K905-0266 TKI treatment is correlated with increase of thyroid specific genes, such as TPO, TSHR, Tg, TTF-1 and Pax-8, as well as NIS.

### Effect of K905-0266 TKI treatment on RAI accumulation and its cytotoxicity in 8505C cells

**Figure 5:**
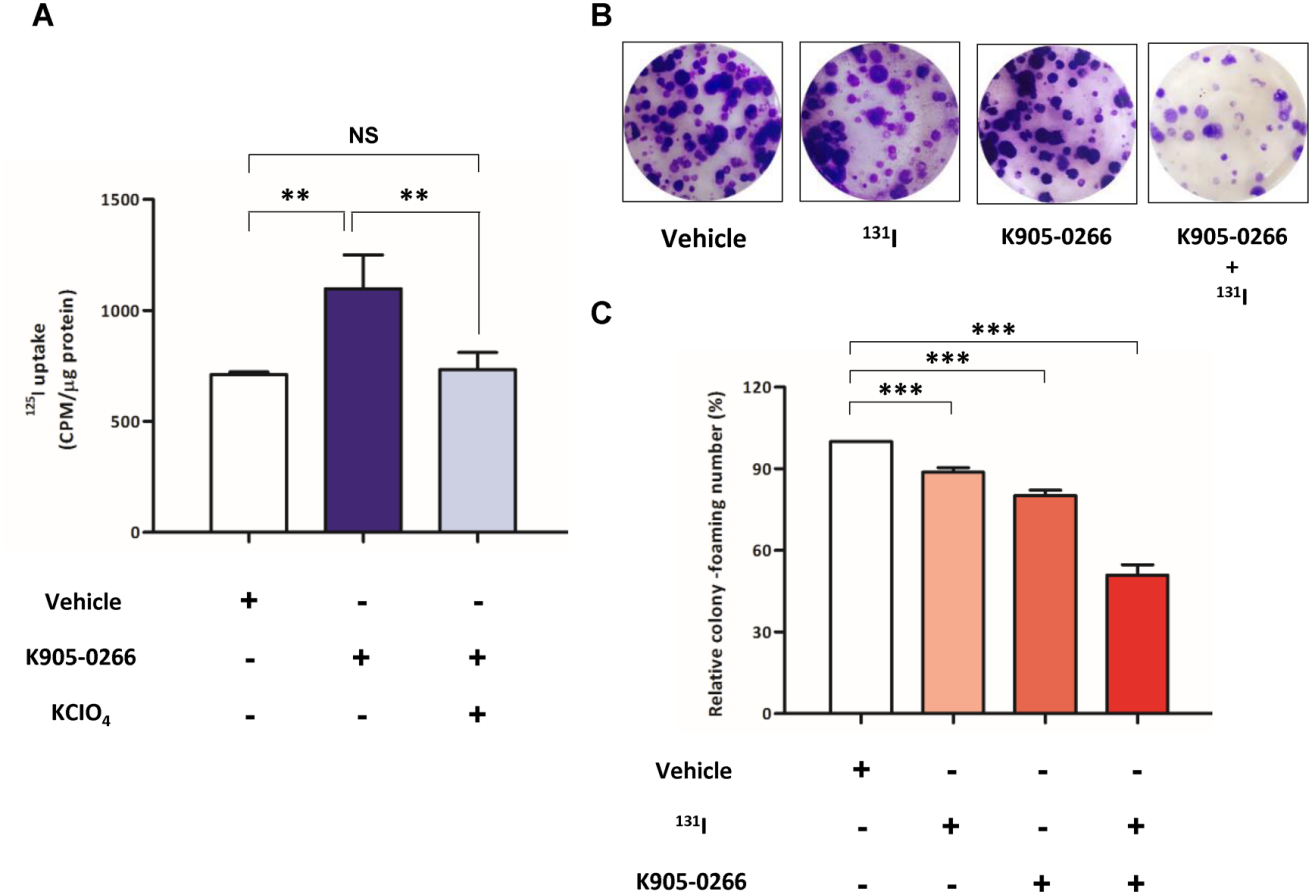
Verification of iodide accumulation ability and cell cytotoxicity by RAI **(A)** ^125^I uptake assay for evaluating iodide accumulation. Cells were treated with 12.5 μM K905-0266 TKI for 24 hours, followed by their incubation with 3.7 kBq carrier-free^125^I and 10 μM/L sodium iodide at 37°C for 30 min. The results are expressed as mean ± standard deviation (SD) of experiments performed in triplicates. ^**^P<0.01, NS (not significant) (by Student's *t*-test). **(B)** Representative images from ^131^I clonogenic assay of the four groups. **(C)** ^131^I clonogenic assay for assessing ^131^I-mediated cytotoxicity effects. The results are expressed as mean ± standard deviation (SD) of experiments performed in triplicates. ^***^P<0.001 (by Student's *t*-test).

To examine RAI accumulation by the regulatory function of the endogenous NIS expression, ^125^I uptake study was performed with or without K905-0266 TKI treatment in 8505C cells. As shown Figure 5A, ^125^I uptake was significantly increased in cells treated with K905-0266 TKI compared to the vehicle control. In addition, ^125^I uptake was completely blocked by potassium perchlorate (KCIO_4_)—a competitive inhibitor of iodide transport. To evaluate whether K905-0266 TKI treatment enhances the therapeutic effect of ^131^I, cells were pre-treated with K905-0266 TKI, followed by ^131^I exposure. While K905-0266 TKI or ^131^I alone induced a minimal decrease in the colony-forming ability, the combination of ^131^I and K905-0266 TKI showed a substantial decrease in the colony-forming ability (Figure 5B, 5C).

### Enhancement of NIS expression by K905-0266 TKI treatment as visualised by immunofluorescence imaging

Immunofluorescence imaging was performed to visualise the enhancement in NIS expression following treatment with K905-0266 TKI. The vehicle control group represented faint NIS expression, but the treatment group showed enhanced NIS protein expression in 8505C cells (Supplementary Figure 4).

### Bioluminescence imaging for the evaluation of the increased NIS promoter following K905-0266 TKI treatment in 8505C-PNIS-PCMV tumour xenograft mice models

**Figure 6:**
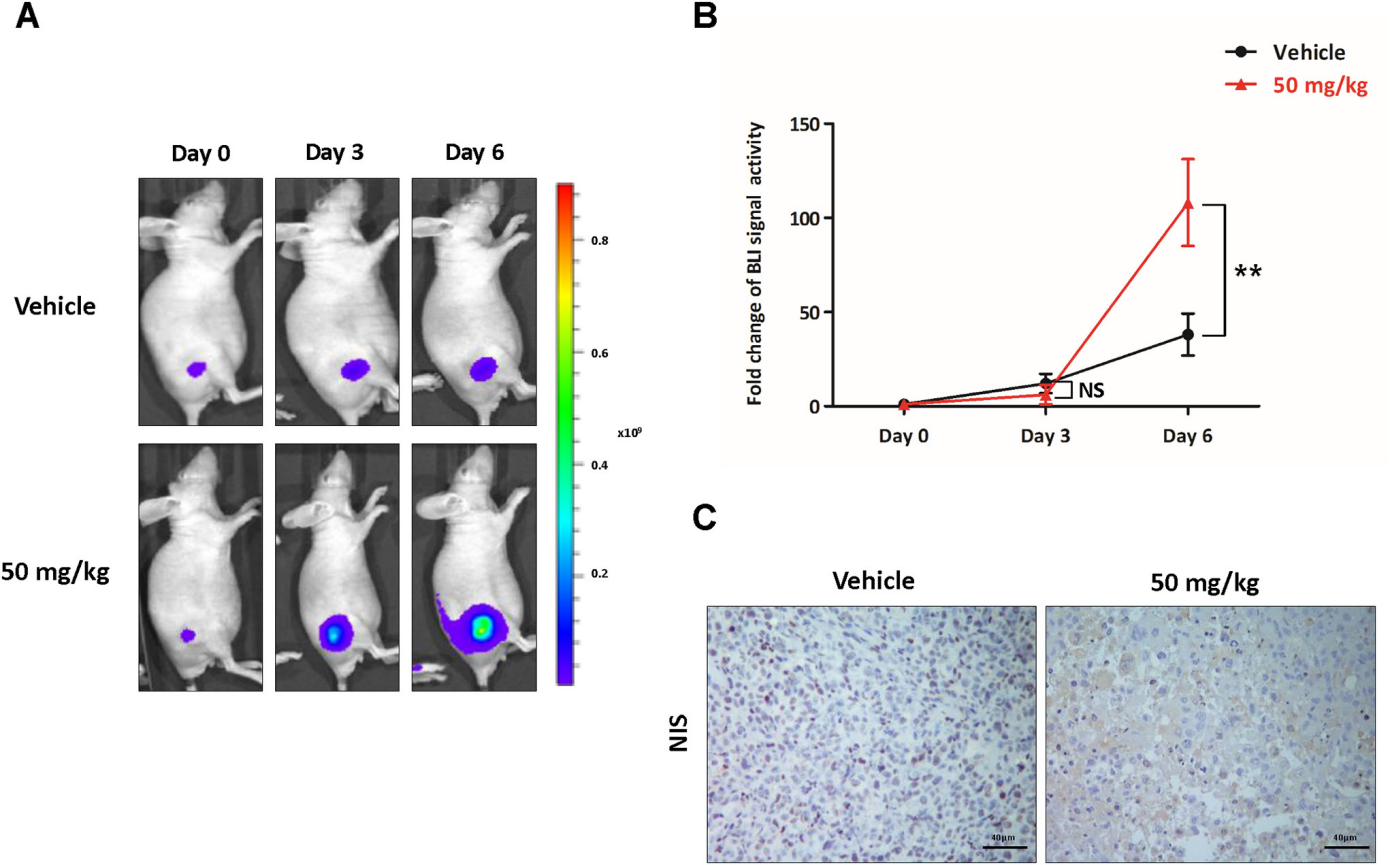
Preclinical evaluation of the functional NIS augmentation following K905-0266 TKI treatment **(A)** Representative images from BLI in 8505C-PNIS-PCMV tumour xenograft mice models. Following treatment with K905-0266 TKI, BLI was evaluated with Fluc on day 0, 3, and 6. **(B)** Graph illustrates quantification of BLI signal activity with Fluc and Rluc photon flux. Data are expressed as the mean ± standard deviation (SD). ^**^P<0.01, NS (not significant) (by Student's *t*-test). **(C)** Representative NIS staining images from 8505C-PNIS-PCMV tumour xenograft mice models. The tumours were excised from the sacrificed mice and subjected to immunohistochemistry for NIS.

To visualise the increase in the expression of NIS following K905-0266 TKI treatment, BLI of Fluc was monitored in 8505C-PNIS-PCMV tumour xenograft mice models based on the *in vitro* experiments. Tumour xenograft mice models were established on day 14 after injection of the cells into the right flank of nude mice. In comparison with the vehicle group, those treated with 50 mg/kg K905-0266 TKI showed a gradual increase in Fluc activity (Figure 6A). Quantitative analyses demonstrated that the tracer accumulation was higher in the group treated with 50 mg/kg K905-0266 TKI than in the vehicle group (12.07±5.13, 38.03±11.09, 6.12±5.08, and 114.77±34.64 for vehicle at day 0, vehicle at day 6, 50 mg/kg K905-0266 TKI at day 0, and 50 mg/kg K905-0266 TKI at day 6, respectively; Figure 6B). The uptick pattern observed for the vehicle group may be attributed to the tumour growth. At the end of the *in vivo* experiment, mice were sacrificed and their tumours excised. Immunohistochemistry analysis showed an increase in NIS expression in 50 mg/kg K905-0266 TKI treatment group, while the expression of NIS was low in the vehicle group (Figure 6C).

### Single and combination therapy with ^131^I and K905-0266 TKI in 8505C-PNIS-PCMV tumour xenograft mice models

**Figure 7:**
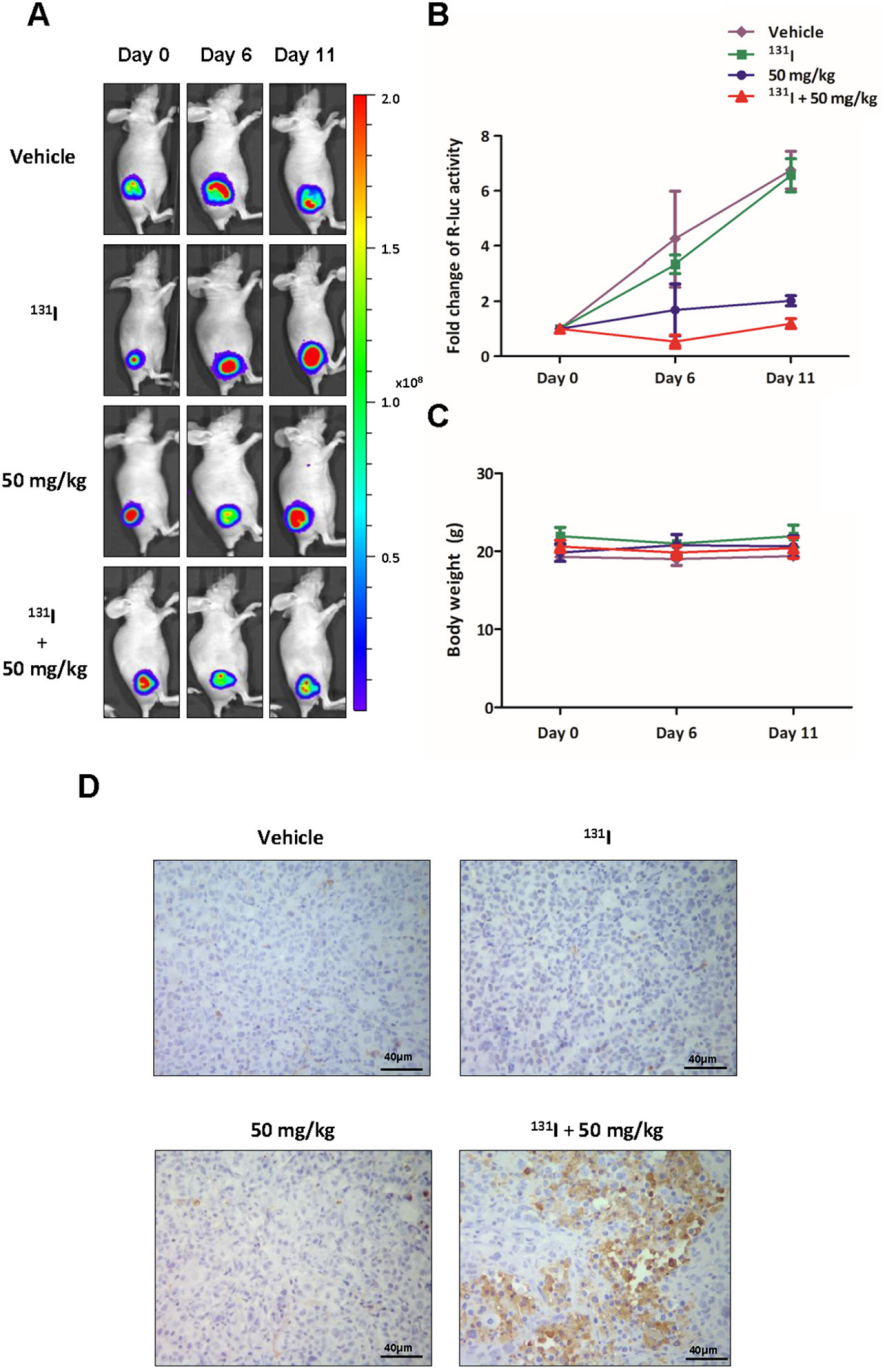
BLI to monitor the therapeutic effects of the combination of ^131^I and K905-0266 TKI in 8505C-PNIS-PCMV tumour xenograft mice models **(A)** Representative Rluc BLI images of 8505C-PNIS-PCMV tumour xenograft mice models with or without administration of ^131^I or K905-0266 TKI. The mice bearing 8505C-PNIS-PCMV cells were divided into four groups: vehicle group, ^131^I treatment group, K905-0266 TKI treatment group, and ^131^I and K905-0266 TKI combination treatment group. BLI with Rluc was acquired on day 0, 6, and 11. **(B)** Graph illustrates fold change in Rluc photon flux. **(C)** The body weight (g) of mice was measured. **(D)** Representative cleaved caspase-3 staining images indicated immunohistochemistry analysis of cleaved caspase-3 from 8505C-PNIS-PCMV tumour xenograft mice models. The tumours were excised from the sacrificed mice and stained for cleaved caspase-3. Scale bars, 40 μm.

The therapeutic effect was assessed by Rluc BLI. The vehicle and ^131^I group showed a continuous increase in Rluc signal intensity at day 6 and 11, while 50 mg/kg K905-0266 TKI treatment group showed retarded tumour growth. However, the combination therapy with ^131^I and 50 mg/kg K905-0266 TKI showed a decrease in Rluc signal intensity at day 6; the signal intensity was significantly lower as compared with other groups (Figure 7A, 7B). The body weight of mice was measured during the *in vivo* experiment. All groups showed no difference in the body weight (Figure 7C). At the end of the *in vivo* experiment, mice were sacrificed and their tumours excised for immunohistochemistry analysis. The results revealed that the level of cleaved caspase-3, which represents the apoptotic area, was highest in the combination treatment group as compared with other groups (Figure 7D).

## DISCUSSION

In the present study, we successfully developed and employed a novel high-throughput NIS enhancer screening platform using dual reporter gene system to screen potent TKIs affecting NIS promoter enhancement in ATC cells. K905-0266 TKI, which was selected as a hit-compound from high-throughput NIS enhancer screening platform, increased NIS promoter activity and enhanced the expression of endogenous NIS and other proteins (Tg, TPO, Pax-8, TSHR, and TTF-1) related to iodide metabolism. Moreover, the suppression of phosphorylated-ERK by K905-0266 TKI may be the mechanism underlying the enhanced expression of endogenous NIS protein, which increased ^125^I uptake *in vitro* and *in vivo*. In addition, K905-0266 TKI pre-treatment enhanced the cytotoxicity of ^131^I therapy against ATC *in vitro* and *in vivo*.

The establishment of a well-designed HTS technology is an important element to identify hit-compounds, which inhibit some specific signal transduction pathways or molecules of interest. HTS technology has been recently used as a key strategy for drug discovery against specific targets [21]. Bioluminescence reporter gene systems are frequently exploited as indicators of target activity to monitor cellular events related to gene expression, regulation, and signal transduction for preclinical applications [23, 24]. Luciferase is a generic term for bioluminescence proteins derived from various sources such as bacteria, marine crustaceans, fish, or terrestrial insects [25]. Luciferase generates visible light through enzymatic reactions with substrates. The bioluminescence reporter gene system offers advantages such as high specificity, low background signal, signal amplification, reliability, convenience, cost-effectiveness, and reproducibility [26]. Therefore, bioluminescence reporter gene systems are appropriate for *in vitro* and *in vivo* preclinical studies. Fluc and Rluc are known as outstanding reporter genes for evaluating the expression of genes, as these lack post-translational modifications, rapidly react with the substrates, and are absent in mammalian cells. These properties of Fluc and Rluc make the luciferase-based bioluminescence reporter gene system an excellent marker for the evaluation of promoter functions in cells [27]. Hence, we designed the dual reporter gene system with Fluc and Rluc to screen an effective NIS enhancer among numerous TKI candidates. We employed the dual reporter gene system in a single vector instead of co-transfection with two vectors, as co-transfection with two vectors poses following disadvantages: each vector may have a different size and the two vectors may not express with the same efficiency in each cell. The dual reporter gene system was designed with a reporter gene system driven by a bidirectional promoter to avoid interference from each other. In this study, we successfully established a novel high-throughput NIS enhancer screening platform with dual reporter gene system using NIS and CMV promoters. The activity of NIS promoter was confirmed through the signal of Fluc, which was placed downstream of NIS promoter. In addition, the activity of CMV promoter, indicative of cell viability, was monitored through the signal of Rluc, which was placed downstream of CMV promoter. As CMV promoter is constitutively expressed in cells, it was used to predict cell viability. K905-0266 TKI as NIS enhancer was selected with high-throughput NIS enhancer screening platform using dual reporter gene system. High-throughput NIS enhancer screening platform offers several benefits for the screening of NIS enhancers as follows: 1) It can monitor NIS promoter activity as well as cell viability in same plates and same animals. 2) Many candidates can be screened at once. 3) It can be monitored in real-time. 3) It is time- and cost-effective. Usually, general HTS system can be applied to non-transfected cells. However, our high-throughput NIS enhancer screening platform system have limitation point that can be selected the hit-compounds using transfected target cells by transfecting the plasmid vector including NIS promoter. The solution of these limitations should be reflected in future experiments.

The genetic alterations of thyroid cancers may cause constitutive activation of MAPK pathway, which may result in the inhibition of iodide-handling genes. ATC is RAI unresponsiveness to most conventional treatments and the median survival of patients with ATC is approximately 6 months [28], highlighting the need for new treatment strategies for patients with ATC. It is known that 45% of ATCs show BRAF^V600E^ mutation and 24% display RAS mutation [29]. The expression of endogenous NIS is suppressed by genetic mutations, which may induce resistance to RAI therapy owing to the activation of MAPK pathway. Therefore, TKIs may serve as good candidates for re-differentiation of ATC. Recent preclinical and clinical studies showed that the blockade of MAPK pathway by TKI treatment is one of the promising strategies to restore RAI avidity in RAI-refractory thyroid cancers [13, 30]. Our results demonstrate that the level of phosphorylated ERK was considerably inhibited by K905-0266 TKI treatment.

The success of RAI therapy primarily relies on RAI accumulation in thyroid cancer. The re-differentiation and re-induction of endogenous NIS expression in response to RAI therapy may be one of the promising therapeutic strategies against thyroid cancers. We evaluated *in vitro* effect of K905-0266 TKI pre-treatment on ^125^I uptake capacity and ^131^I cytotoxicity against ATC. K905-0266 TKI treatment restored radioiodine avidity in ATC cells and reverted RAI unresponsiveness ATC cells into RAI-sensitive ATC cells. Furthermore, *in vivo* experiments demonstrated that K905-0266 TKI treatment enhances NIS promoter activity in ATC tumour xenograft mice models. In addition, RAI treatment followed by K905-0266 TKI administration successfully inhibited the growth of ATC tumour, as demonstrated using BLI with Rluc. It should be also noted that the single treatment of K905-0266 TKI showed the ability to impede tumour growth. Therefore, single treatment of K905-0266 TKI may be applied for ATC chemotherapy.

In this study, a novel high-throughput NIS enhancer screening platform using dual reporter gene system, which screened NIS enhancers, was developed. K905-0266 TKI, identified using the high-throughput NIS enhancer screening platform, increased NIS expression in ATC and reverted to RAI-sensitive ATC by re-differentiation. The study using high-throughput NIS enhancer screening platform may be applied to various thyroid cancer cells to find new hit-compounds that increase the expression of endogenous NIS.

## MATERIALS AND METHODS

### Cell culture

The human ATC cell line 8505C was purchased from Deutsche Sammlung von Mikroorganismen und Zellkulturen (DSMZ) and maintained in Roswell Park Memorial Institute (RPMI)-1640 medium (HyClone, Logan, UT, USA) supplemented with 10% fetal bovine serum (FBS; Gibco, Grand Island, NY, USA) and 1% penicillin-streptomycin (HyClone) in a humidified incubator at 37°C and 5% CO_2_.

### Construction of plasmid

A pNIS-FL2-TurboFP635-pCMV-Rluc plasmid DNA expressing the reporter gene driven by dual promoters was established by Cosmo Genetech Co. Ltd. (South Korea) using pNIS-FL2-TurboFP635 and pcDNA3.1/Hygro (+) vectors. pNIS-FL2-TurboFP635 vector was a kind gift from Dr. Abhijit De, ACTREC, India. pcDNA3.1/Hygro (+) vector was purchased from Invitrogen (Carlsbad, CA, USA). For monitoring NIS promoter/enhancer, the downstream region (clockwise) of NIS promoter included the gene for firefly luciferase (Fluc) and TurboFP635. Cytomegalovirus promoter with *Renilla* luciferase (Rluc) gene was placed in the opposite direction (anti-clockwise) of NIS promoter region to evaluate cell viability. The details of vector diagram are indicated in Supplementary Figure 1.

### Establishment of stable cell line

The pNIS-FL2-TurboFP635-pCMV-Rluc plasmid DNA was transfected in 8505C cells using Fu-GENE HD reagent, as per the manufacturer's instructions (Promega, Madison, WI, USA). The established stable cell line having the dual reporter gene system is termed as “8505C-PNIS-PCMV” cell line.

### High-throughput NIS enhancer screening platform for excavating NIS enhancer from TKI libraries in 8505C-PNIS-PCMV cells

To excavate the NIS enhancer by examining transcriptional regulation of NIS promoter by high-throughput NIS enhancer screening platform, 8505C-PNIS-PCMV cells were treated with a range of TKIs for 24 hours. Fluc activity was measured using IVIS Lumina III (Perkin-Elmer, Wellesley, MA, USA) by adding 150 μg/mL d-luciferin, while Rluc activity was measured using IVIS Lumina III by adding 10 μg/mL h-coelenterazine. The kinase library used in this study was a kind gift provided by the Korea Chemical Bank (http://www.chembank.org/) of Korea Research Institute of Chemical Technology. K905-0266 TKI was selected among TKI candidates as a hit-compound for both *in vitro* and *in vivo* experiments. K905-0266 TKI was purchased from ChemDiv (San Diego, CA, USA) and dissolved in dimethyl sulfoxide (DMSO) and stored at −80°C. The information about K905-0266 TKI is shown in Supplementary Figure 3.

### Protein extraction

For the extraction of total protein, 8505C cells were cultured with K905-0266 TKI for 24 hours in a CO_2_ incubator at 37°C and washed twice with chilled phosphate-buffered saline (PBS). Cell pellets were lysed using radioimmunoprecipitation assay (RIPA) buffer (Thermo Fisher Scientific, Rockford, IL, USA) containing protease and phosphatase inhibitor cocktail kit (Thermo Fisher Scientific). The lysed cells were briefly vortexed (thrice) at intervals and subsequently centrifuged at 13,000 ×*g* at 4°C. Membrane and cytoplasm proteins were extracted from the soluble protein fraction using Mem-PER™ Plus kit (Thermo scientific) according to the instructions of the manufacturer. Briefly, the collected cell pellet was washed with cell wash solution and centrifuged twice at 300 ×*g* for 5 minutes. The supernatant was discarded and the cell pellet was treated with a permeabilization buffer. To obtain a homogeneous cell suspension, the cell pellet was vortexed and incubated for 10 minutes at 4°C with continuous mixing. Following incubation, the cell pellet was centrifuged and the supernatant containing cytoplasm proteins extracted. The remnant pellet was treated with the solubilisation buffer for 10 minutes at 4°C with continuous mixing. After centrifugation at 300 ×*g* for 5 minutes, membrane proteins were transferred into a new tube. The protein sample was quantified with the bicinchoninic acid (BCA) protein assay kit (Thermo Fisher Scientific).

### Western blot

Equal amounts of proteins were loaded onto 10% sodium dodecyl sulfate polyacrylamide gel electrophoresis and transferred onto PVDF membranes (Millipore, Billerica, MA, USA). The membrane was blocked with 3% BSA in Tris-buffered saline (TBS) containing Tween-20 (TBS-T) for an hour and probed with the respective primary antibodies in 0.5% BSA overnight at 4°C. Following incubation, the membrane was probed with horseradish peroxidase (HRP)-conjugated secondary antibodies for an hour at room temperature. The membrane was washed thrice with TBS-T and the signal was visualised using an enhanced chemiluminescence (ECL) detection reagent (GE Healthcare Life Sciences, Pittsburgh, PA, USA). The bands were detected using Fusion FX chemiluminescence analyzer system (Vilber lourmat, Marne-la-Vallée, France), according to manufacturer's protocol. The primary antibodies used were as follows: NIS (Thermo Fisher Scientific; working dilution 1:2500), phospho-p44/42 MAPK (phosphorylated extracellular-signal-regulated kinase [pERK1/2]) (Cell Signaling; dilution 1:2500), p44/42 MAPK (Total ERK1/2) (Cell Signaling; dilution 1:2500), phospho-Akt (Cell Signaling; dilution 1:2500), Akt (Cell Signaling; dilution 1:2500), thyroglobulin (Tg, Santa Cruz Biotechnology; dilution 1:2000), thyroid-stimulating hormone receptor (TSHR; Santa Cruz Biotechnology; dilution 1:2000), thyroperoxidase (TPO, Santa Cruz Biotechnology; dilution 1:2000), thyroid transcription factor-1 (TTF-1; Santa Cruz Biotechnology; dilution 1:2000), paired-box gene 8 (Pax-8; Santa Cruz Biotechnology; dilution 1:2000), Firefly luciferase (Promega; dilution 1:5000), *Renilla* luciferase (Abcam; dilution 1:5000), glyceraldehyde 3-phosphate dehydrogenase (GAPDH; Santa Cruz Biotechnology; dilution 1:5000), and β-actin (Santa Cruz Biotechnology; dilution 1:5000). HRP-conjugated secondary antibodies used were as follows: anti-mouse (Cell Signaling) and anti-rabbit (Cell Signaling).

### *In vitro* ^125^I uptake assay

To study ^125^I uptake, 8505C cells (1.25×10^5^) were seeded in 24-well plates for 24 hours and incubated with K905-0266 TKI for 24 hours at 37°C. After incubation, the medium was aspirated and 8505C cells were washed with Hank's balanced salt solution (HBSS) containing 0.5% BSA (bHBSS). The cells were incubated with 500 μL bHBSS, 3.7 kBq carrier-free ^125^I (Perkin-Elmer, Waltham, MA, USA), and 10 μM/L sodium iodide (NaI, specific activity of 740 MBq/mM) at 37°C for 30 min. Following incubation, cells were washed twice with chilled bHBSS and lysed with 500 μL of 2% SDS. Radioactivity was measured using a Cobra II gamma-counter (Canberra Packard, Mississauga, Canada). The uptake values were normalised with the amount of total protein determined by BCA protein assay kit (Thermo Fisher Scientific).

### ^131^I clonogenic assay

For ^131^I clonogenic assay, 8505C (4×10^5^) cells were seeded in a six-well plate and incubated with K905-0266 TKI for 24 hours in a humidified incubator at 37°C and 5% CO_2_. The medium was aspirated and cells were rinsed twice with bHBSS. Thereafter, the cells were incubated with or without 50 μCi/mL ^131^I (KIRAMS, Seoul, Korea) supplemented with 30 μM NaI for 7 hours at 37°C in a 5% CO_2_ humidified atmosphere. After incubation, cells were washed twice with bHBSS and re-seeded at a density of 1,000 cells/well in a new six-well plate for 7 days to allow colony formation in a humidified incubator at 37°C and 5% CO_2_. At day 7, the medium was removed and cells were rinsed twice with PBS. The fixation buffer containing 1:7 acetic acid:methanol was added to the wells and the plate incubated at room temperature for 5 minutes to fix colonies. The fixed colonies were stained with 0.05% crystal violet for an hour and immersed in tap water to rinse off crystal violet. The colonies of each group with over 50 cells were counted. Percentage inhibition was calculated as the number of colonies formed under the treatment conditions relative to the vehicle control.

### Immunofluorescence microscopy

8505C cells (2×10^5^) were seeded and treated with K905-0266 TKI for 24 hours. Following incubation, cells were fixed with 4% paraformaldehyde for 30 minutes at room temperature and rinsed thrice with PBS for 10 minutes. Cells were permeabilized with 0.1% Triton in PBS for 10 minutes at room temperature and quenched with 50 mM NH_4_Cl in PBS for 10 minutes at room temperature, followed by three washes with PBS for 5 minutes. The cells were blocked with 5% BSA in PBS and overnight incubated with anti-NIS (Abcam, Cambridge, UK) primary antibody (1:50 ratio) at 4°C. Following treatment, cells were washed thrice with PBS for 10 minutes and probed with Alexa-Fluor 488-conjugated secondary antibody (Thermo Fisher Scientific) at 1:300 dilution for an hour. The cells were mounted on a coverslip using Vecta mounting medium containing 4′,6-diamidino-2-phenylindole (DAPI; Vector Laboratories, Burlingame, CA, USA). NIS staining was observed by confocal laser microscopy (Zeiss, LSM 5 exciter, Germany).

### Animal models

Female Balb/c nude mice, 5.5-week old with an average weight of 18.9±0.37 g (mean ± standard deviation [SD]), were purchased (Hamamatsu, Shizuoka, Japan). The mice were maintained under specific pathogen-free conditions for a week to adapt to the experimental conditions before starting the experiment. The animals were maintained at room temperature (20–25°C) and 40–70% relative humidity. To establish tumour xenograft mice models, 8505C-PNIS-PCMV cells (5×10^6^) were mixed with Matrigel (Corning, Bedford, MA, USA) at 1:1 ratio and subcutaneously injected into right flank region.

### *In vivo* bioluminescence imaging

To assess NIS promoter enhancement by K905-0266 TKI treatment, mice were divided into two groups: the vehicle group (n=5) and treatment group (50 mg/kg K905-0266 TKI, n=5). After establishment of the tumour xenograft mice models, 50 mg/kg K905-0266 TKI was administered intraperitoneally every day for 5 days. On day 0 and 6, Rluc BLI was carried out using IVIS Lumina III of the intravenous administration of 15 μg/mL h-coelenterazine. Fluc BLI for an intraperitoneal injection of 150 μg/mL d-luciferin was also carried out. For assessing the therapeutic effectiveness of treatments, mice were divided into four groups: vehicle group (n=5), ^131^I group (1 mCi Na-^131^I on day 2 intraperitoneally, n=5), K905-0266 TKI treatment group (50 mg/kg for 5 days intraperitoneally, n=5), and combination group (^131^I and K905-0266 TKI; intraperitoneal injection of 50 mg/kg K905-0266 TKI for 5 days followed by intravenous injection of 1 mCi Na-^131^I on day 2, n=5). The growth of tumour was monitored by Rluc BLI, which was measured every 5 days by intravenous administration of 15 μg/mL h-coelenterazine.

### Immunohistochemistry

The mice were sacrificed by cervical vertebrae dislocation at the end of the experiment. The tumours were removed and fixed overnight with 4% formalin. The specimens were embedded in paraffin, sectioned into 4-μm thickness, and mounted on slides. The specimen sections on slides were de-paraffinised and stained using hematoxylin and eosin (H&E). For immunohistochemical staining, NIS (Thermo Fisher Scientific; working dilution 1:200) and cleaved caspase-3 (Cell Signaling; dilution 1:200) were used.

### Ethical statement

All described procedures were reviewed and approved by Kyungpook National University Animal Care and Use Committee (KNU-2012-43) and performed in accordance with the Guiding Principles for the Care and Use of Laboratory Animals.

### Statistical analysis

All data are expressed as mean ± SD. Data from two groups were statistically analysed by Student's *t*-test using GraphPad Prism 5 software version 5.01 (GraphPad Software, Inc. La Jolla, CA, USA). A value of *P* less than 0.05 was considered statistically significant. Error bars represented standard deviations.

## CONCLUSION

In present study, a novel high-throughput NIS enhancer screening platform with dual reporter gene system was successfully developed for screening NIS enhancers. Using this platform, K905-0266 TKI was identified as a potent NIS enhancer and its function was verified both *in vitro* and *in vivo*. High-throughput NIS enhancer screening platform may be used to discover effective NIS enhancers, while K905-0266 TKI may serve as the potent NIS enhancer to treat thyroid cancers in combination with RAI.

## SUPPLEMENTARY MATERIALS AND FIGURES


