# Supplementary material for: Reverting iodine avidity of radioactive-iodine refractory thyroid cancer with a new tyrosine kinase inhibitor (K905-0266) excavated by high-throughput NIS (sodium iodide symporter) enhancer screening platform using dual reporter gene system
Source: Oncotarget. 2018 Jan 11;9(6):7075–87. doi: 10.18632/oncotarget.24159 (PMC5805537; doi:10.18632/oncotarget.24159)
Supplement: Supplementary file 1 [file oncotarget-09-7075-s001.pdf]

Reverting iodine avidity of radioactive-iodine refractory thyroid cancer with a new tyrosine kinase inhibitor (K905-0266) excavated by high-throughput NIS (sodium iodide symporter) enhancer screening platform using dual reporter gene system

SUPPLEMENTARY MATERIALS

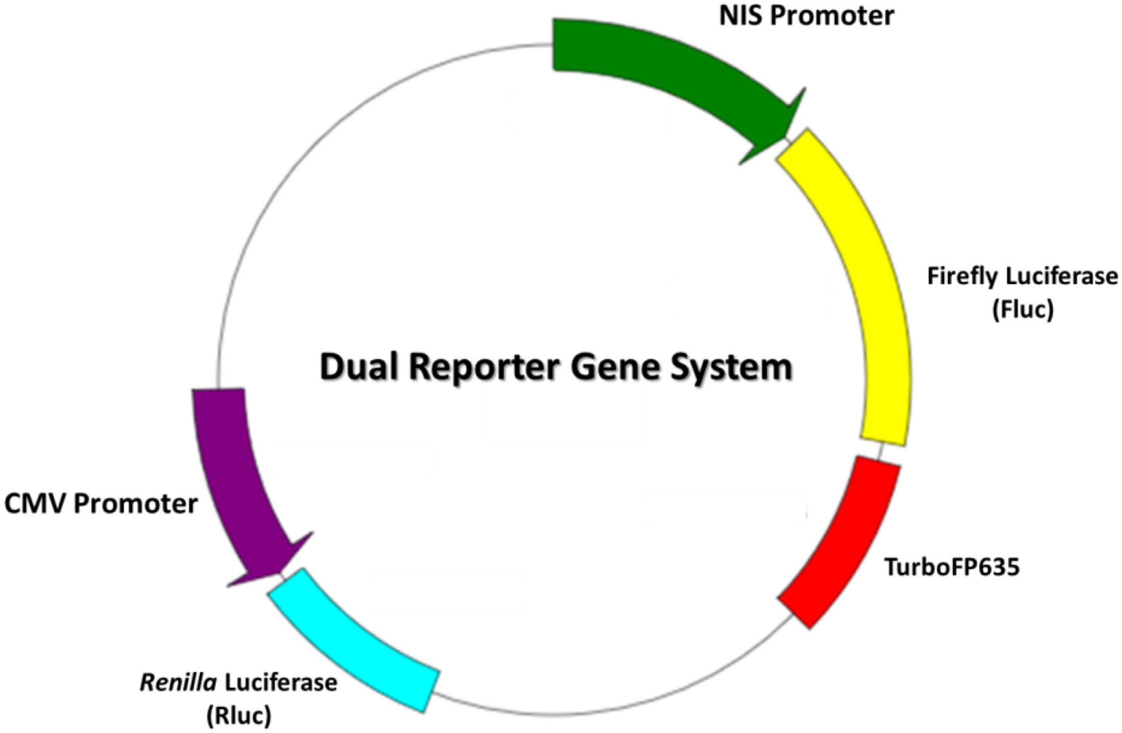

Supplementary Figure 1: Diagram of the vector expressing dual reporter gene system, which contained specific promoters (NIS and CMV promoter).

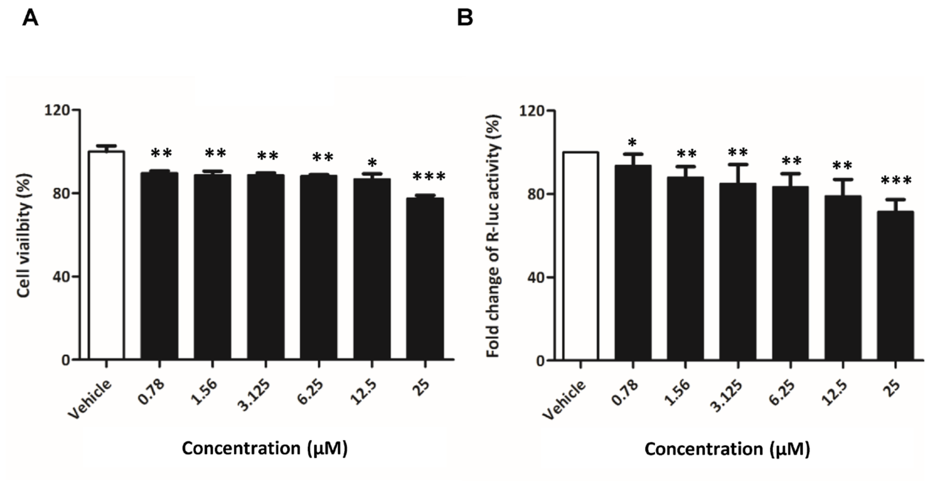

Supplementary Figure 2: Correlation between CCK-8 assay and Rluc activity showing cell viability. 8505C-PNIS-PCMV cells were treated with K905-0266 TKI at increasing concentrations for 24 hours. The graphs illustrate **(A)** CCK-8 assay and **(B)** Rluc activity. Data are expressed as the mean ± standard deviation (SD), $^{*}P<0.05$, $^{**}P<0.01$, $^{***}P<0.001$ (by Student's t-test).

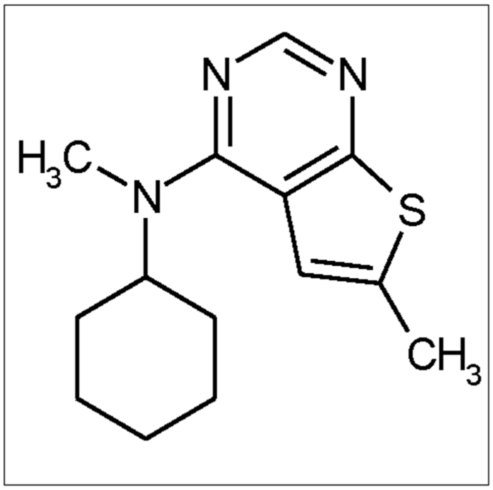

Product name	Cyclohexyl-methyl-(6-methyl-thieno[2, 3-d]pyrimidin-4-yl)-amine
Synonyms	K905-0266
Molecular formula	$C_{14}H_{19}N_3S$
Molecular weight	261.39g/mol.

Supplementary Figure 3: Information of K905-0266 TKI.

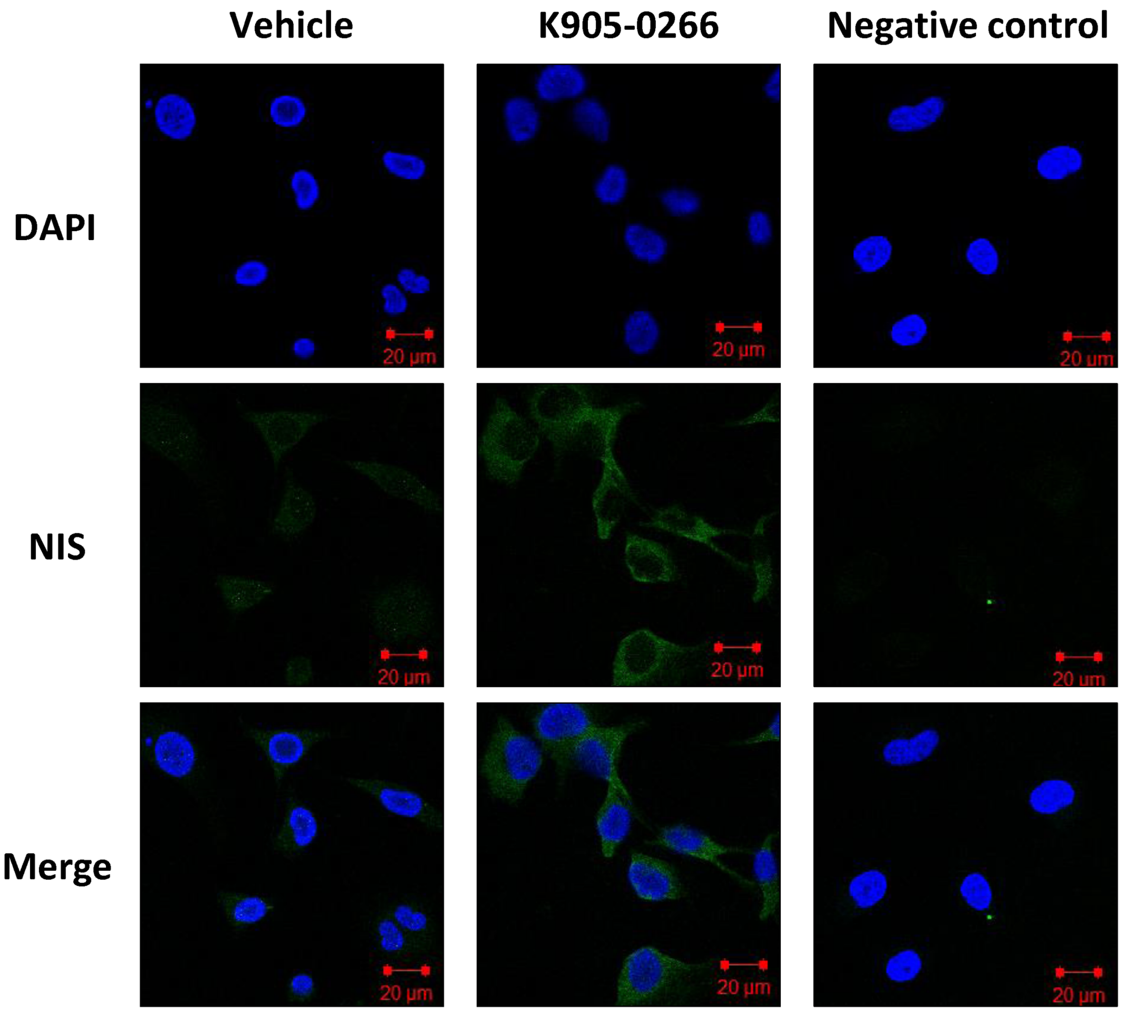

Supplementary Figure 4: Immunofluorescence analysis of the endogenous NIS expression. Representative confocal images of NIS expression in 8505C cells treated with or without K905-0266 TKI. DAPI and NIS were visualized in blue and green, respectively. The negative control was processed only with the secondary antibody. The scale bars (red) represent 20 µm.
